# A treatment-response framework for interpreting endovascular thrombectomy evidence: established practice, boundary expansion and unresolved frontiers

**DOI:** 10.3389/fneur.2026.1951552

**Published:** 2026-09-22

**Authors:** Naibijiang Abuliezi, Jiale Zhao, Yihan Sun, Tayier Tuersong, Xiaoyi Dun, Qinfen Wu

**Affiliations:** 1 The Second Affiliated Hospital of Xinjiang Medical University, Urumqi, China; 2 Department of Pharmacy, The Second Affiliated Hospital of Xinjiang Medical University, Urumqi, China; 3 The Fifth Affiliated Hospital of Xinjiang Medical University, Urumqi, China; 4 Xinjiang Key Laboratory of Neurological Disorder Research (Lab No.: XJDX1711), Urumqi, China; 5 Xinjiang Clinical Research Center for Neurological Diseases, Urumqi, China

**Keywords:** acute ischaemic stroke, basilar artery occlusion, endovascular thrombectomy, large-core infarction, large-vessel occlusion, late-window stroke, medium-vessel occlusion, treatment-effect heterogeneity

## Abstract

This structured narrative review evaluates endovascular thrombectomy (EVT) through an author-developed, population-level treatment–response framework. In this framework, treatment response is the causal contrast between EVT plus best medical care and best medical care alone on a specified patient-centered outcome; it is not the outcome observed after EVT, and it cannot be observed for an individual patient because the untreated counterfactual is unknown. The framework is an evidence-interpretation tool, not a statistical estimator, a validated decision aid, or an individual prediction model. It separates four clinically actionable questions: first, trial eligibility—does the patient resemble a population in which EVT was tested; second, baseline prognosis—which features predict outcome under either strategy; third, procedural feasibility and safety—can reperfusion be achieved promptly at acceptable risk; and fourth, treatment-effect modification—does randomized evidence support a different relative or absolute effect across levels of a baseline variable? We conducted targeted, purposive searches across PubMed/MEDLINE, supplemented by backward and forward citation tracking, to identify pivotal randomized trials, individual participant data meta-analyses, population-specific guidelines, and directly relevant secondary analyses through 15 August 2026. Randomized evidence supports EVT for proximal anterior-circulation large-vessel occlusion in the early window, selected 6–24 h anterior-circulation populations, trial-defined large-core infarction, and selected moderate-to-severe basilar artery occlusion. Dedicated randomized evidence remains absent beyond 24 h and incomplete for large-vessel occlusion with a low National Institutes of Health Stroke Scale (NIHSS) score. ESCAPE-MeVO, DISTAL, and DISCOUNT did not demonstrate average functional benefit in broad medium/distal-vessel populations, whereas ORIENTAL-MeVO reported benefit in an enriched population with NIHSS at least 6; these results should not be generalized across all distal occlusions. Failure to detect an interaction does not establish homogeneous effects because interactions are outcome- and scale-specific, and most EVT trials are underpowered to detect them. Poor outcomes after successful reperfusion are descriptive of post-treatment states, not proof that EVT was futile. This framework clarifies what randomized evidence establishes, what remains prognostic or procedural, and where prospective testing is still required.

## 1 Introduction

Stroke remains a major cause of death and disability. In the Global Burden of Disease 2021 analysis, stroke accounted for 11.9 million incident events, 93.8 million survivors, 7.3 million deaths, and 160.5 million disability-adjusted life years in 2021. Ischemic stroke represented nearly two-thirds of incident strokes (1). Proximal anterior-circulation large-vessel occlusion (LVO) represents a key setting where rapid mechanical reperfusion can change 90-day disability outcomes, as demonstrated by the pivotal early endovascular thrombectomy (EVT) trials and their individual-participant-data synthesis (2–7).

The pivotal early-window EVT trials established a causal population-average treatment effect: in selected patients with proximal anterior-circulation LVO, EVT plus best medical care improved 90-day disability compared to best medical care alone (3–7). Nevertheless, trial eligibility, baseline prognosis, procedural success, and causal benefit answer different questions. An eligible patient may still have a poor prognosis; a technically accessible clot may not be treated without clinically important delay or harm; and successful reperfusion or an observed post-EVT outcome does not reveal the untreated counterfactual (8, 9).

Earlier imaging-selection studies helped define both the rationale and the limits of mismatch-based reperfusion selection: DEFUSE 2 linked target mismatch to response after endovascular reperfusion, whereas MR RESCUE did not show that a penumbral pattern identified benefit with the studied endovascular approach (10, 11). Late-window randomized evidence then made these distinctions more important. DAWN identified a clinical–core mismatch population stratified by age and the National Institutes of Health Stroke Scale (NIHSS) treated at 6–24 h (12), whereas DEFUSE 3 tested perfusion-mismatch selection at 6–16 h (13). AURORA confirmed an average benefit across pooled trial-selected late-window populations (14), and MR CLEAN-LATE demonstrated a pragmatic collateral-based selection pathway at 6–24 h (15). Subsequent randomized controlled trials (RCTs) tested defined large-core populations, with five positive trials and TESLA not meeting its prespecified Bayesian superiority threshold (16–21). ATLAS subsequently pooled individual data from 1,886 participants across all six trials and confirmed a better 90-day modified Rankin Scale (mRS) distribution with EVT [adjusted generalized odds ratio (OR) 1.63, 95% confidence interval (CI) 1.42–1.88] (22). Basilar artery occlusion (BAO) trials were neutral or inconclusive in BEST and BASICS but positive in the more selected ATTENTION and BAOCHE populations (23–26). Contemporary medium/distal-vessel occlusion (MeVO/DMVO) trials were neutral or unfavorable in three broadly defined populations, whereas ORIENTAL-MeVO was positive in an enriched population (27–30).

Existing heterogeneity-of-treatment-effect frameworks, including the Predictive Approaches to Treatment Effect Heterogeneity (PATH) statement, provide statistical principles for examining variation in relative and absolute treatment effects (8). Unlike those statistical frameworks and individualized-treatment-effect models, the present author-developed framework does not estimate treatment effects or claim to predict an individual counterfactual. Its novelty is an EVT-specific conceptual checklist that assigns each clinical, imaging, and procedural variable to its evidentiary role before it is used in a treatment argument. Its distinguishing additions are explicit consideration of trial eligibility and transportability, procedural feasibility and safety, and separation of post-randomization technical measures such as modified Thrombolysis in Cerebral Infarction (mTICI) from causal treatment benefit. We apply the same four questions to each major EVT population and identify where claims are supported by randomized comparisons, where they remain prognostic or procedural, and where credible interaction evidence is absent. The framework has not been validated as a clinical decision aid.

## 2 Methods

### 2.1 Review design and scope

This was a structured, purposive narrative review. The purpose was to identify pivotal and directly relevant evidence for predefined clinical questions, not to provide exhaustive systematic study identification or quantitative pooling. The review addressed early and late anterior-circulation LVO, large-core infarction, BAO, low-NIHSS LVO, MeVO/DMVO, treatment beyond 24 h, and interpretation of poor outcomes after successful reperfusion.

### 2.2 Search strategy and study selection

We searched PubMed/MEDLINE from database inception through 15 August 2026 using a common stroke-and-thrombectomy block combined separately with seven prespecified modules: late-window or imaging selection, large core, BAO, low NIHSS or minor stroke, MeVO/DMVO, reperfusion, no-reflow or historically termed “futile reperfusion,” and workflow or evidence translation. Exact date-stamped PubMed strings used for the revision update are provided in Supplementary Table S1. We supplemented these searches with backward and forward citation tracking from landmark RCTs, individual-participant-data (IPD) meta-analyses, population-specific guidelines, and recent high-quality reviews. Targeted publisher and trial registry searches were conducted to identify online-first reports and ongoing trials, including the final DISCOUNT report published online on 22 July 2026 (29).

Records were evaluated at the title/abstract level and then at the full-text level when they directly informed at least one framework domain: trial eligibility, prognosis, procedural feasibility or safety, average treatment effect, treatment-effect modification, endpoint interpretation, or external-validity boundaries. Primary RCT reports were prioritized. IPD meta-analyses and current guidelines were used to integrate trial families; secondary analyses were retained only for prespecified modifier, endpoint, or implementation questions. Observational studies were labeled as non-randomized and hypothesis-generating. Case reports, purely technical studies without relevance to the review questions, duplicate reports without additional information, and non-English reports without unique landmark evidence were not retained.

### 2.3 Evidence extraction, verification and limitations

One reviewer extracted study population, sample size, time window, selection criteria, intervention and comparator, primary outcome, principal effect estimates, and major safety outcomes into a trial matrix. A second reviewer independently verified these fields and the adjacent claim–citation mapping against the primary report or its indexed abstract. Disagreements about retention, numerical extraction, or wording were resolved by consensus. This process was duplicate verification of the final evidence set, not duplicate screening of every retrieved record.

We did not undertake exhaustive PRISMA screening, calculate a flow diagram, perform a formal risk-of-bias assessment, or conduct a new meta-analysis. The absence of a study from this purposive synthesis should not be interpreted as evidence that it does not exist. Throughout the review, we distinguished prognostic association from randomized treatment interaction and biological plausibility from evidence of efficacy.

## 3 Defining the population-level treatment-response framework

### 3.1 The causal contrast and its limits

For a specified patient-centered endpoint, treatment response means the contrast between the outcome that would occur with EVT plus best medical care and the outcome that would occur with best medical care alone. Only one potential outcome can be observed in an individual. RCTs therefore estimate an average treatment effect in a trial-defined population; they do not reveal whether a particular treated patient personally benefited or create a validated individual response score (8).

This definition separates treatment effect from three clinically important but causally different observations. First, baseline prognosis is the expected outcome under either treatment strategy. Second, post-treatment technical measures such as expanded Thrombolysis in Cerebral Infarction (eTICI) grade or first-pass reperfusion describe procedural performance. Third, the 90-day modified Rankin Scale (mRS) is the observed patient outcome. Neither technical success nor the observed outcome alone identifies the untreated counterfactual (9).

### 3.2 Four clinically actionable questions

The framework asks four questions in sequence (Figure 1).

*Trial eligibility*: Does the patient match a population, time window, and selection rule in which EVT was compared with non-EVT care? Eligibility defines the evidence base; it does not guarantee benefit for every eligible patient.
*Baseline prognosis*: Which pretreatment features predict outcome regardless of assignment? Age, baseline disability, deficit severity, infarct burden, edema, and collateral status may inform prognosis, but a prognostic association is not evidence that EVT benefit differs (2, 8).
*Procedural feasibility and safety*: Can the target be reached and reperfused promptly with an acceptable risk of vessel injury, hemorrhage, embolization, and delay? These factors affect the expected benefit–harm balance and may limit transportability across systems (2–7, 31).
*Treatment-effect modification*: Does randomized evidence show that a baseline variable changes the contrast between EVT and control on a prespecified endpoint and effect scale? A credible claim requires a treatment-by-variable interaction or an appropriately modeled risk-based analysis using randomized data (8).

**Figure 1:**
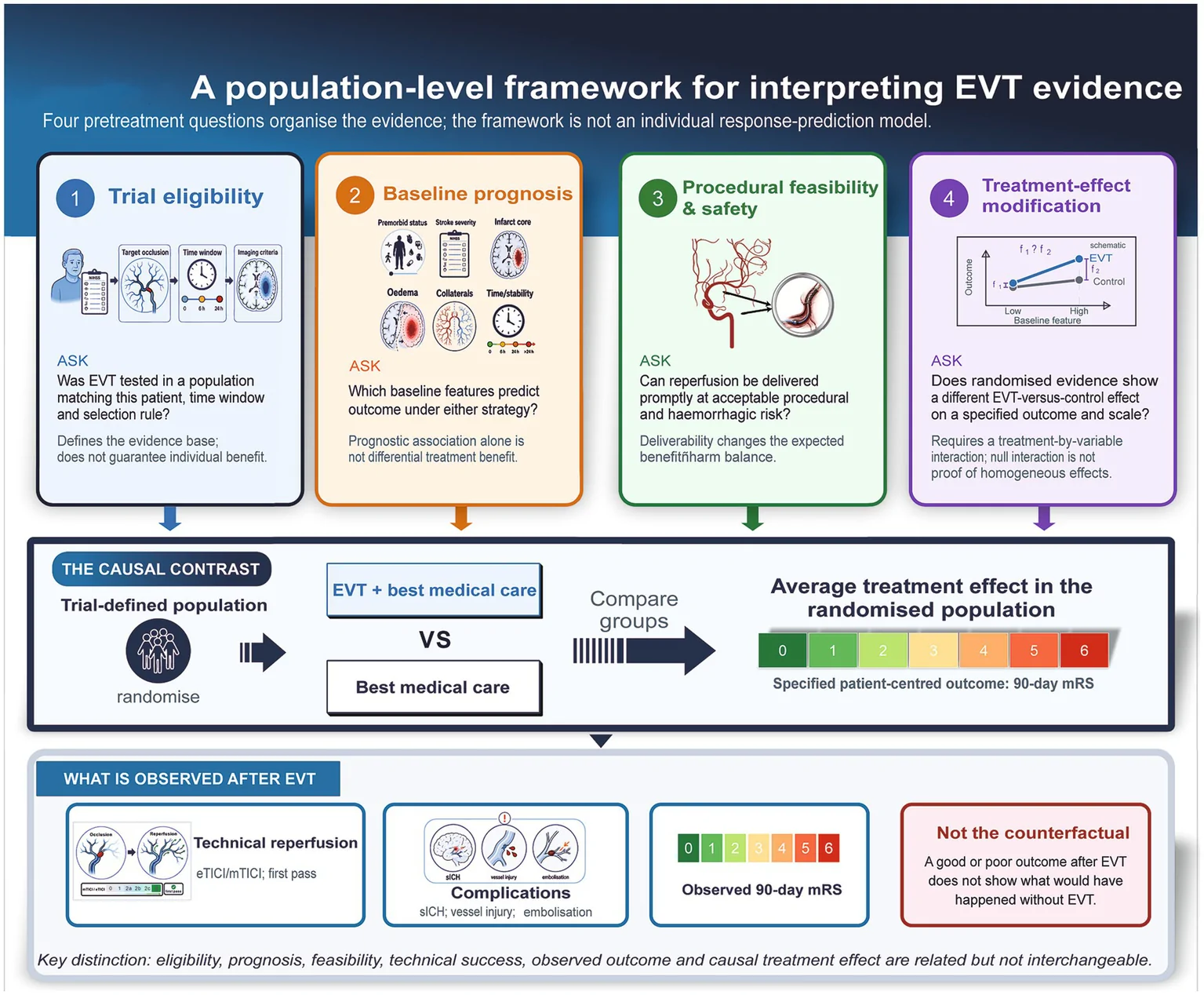
Population-level framework for interpreting EVT evidence. Four pretreatment questions—trial eligibility, baseline prognosis, procedural feasibility and safety, and credible randomised treatment-effect modification—organise the evidence. Randomisation estimates an average treatment effect in a trial-defined population by comparing EVT plus best medical care with best medical care alone (8, 9). Technical reperfusion, complications and the observed 90-day outcome are post-treatment measures; none reveals the untreated counterfactual (63–68). Representative imaging, vascular and device elements are retained as visual anchors but do not carry evidentiary weight. The framework is author-developed, unvalidated and not an individual prediction model. EVT, endovascular thrombectomy; mRS, modified Rankin Scale; RCT, randomised controlled trial.

The fourth question requires particular caution. Interaction is specific to the chosen outcome and effect scale: relative effects can appear similar while absolute benefit differs because baseline risk differs. Most EVT trials were powered for an overall effect, not for interaction testing. Consequently, failure to demonstrate an interaction does not establish homogeneous effects, whereas within-EVT prognostic associations and *post hoc* subgroup differences do not establish modification (8).

### 3.3 Difference from existing precision-medicine frameworks

PATH and related frameworks focus on methods for estimating heterogeneous relative or absolute treatment effects (8). The present framework is complementary and deliberately non-statistical. It begins one step earlier by classifying the evidentiary role and transportability of a variable, and one step later by separating causal effect from post-treatment technical and clinical observations. Its contribution is conceptual and translational: it provides a repeatable EVT-specific structure for reading trials and avoiding category errors at the bedside and in narrative synthesis. It is author-developed and unvalidated; it should not be used as a bedside score. Figure 1 contains the causal framework alone. Figure 2 is a separate factual matrix that reports, for each population, the direction of completed dedicated efficacy RCT evidence, a representative estimate on its original outcome scale, the eligibility boundary actually tested, and the uncertainty remaining beyond direct randomized inference. Rows follow manuscript order; no axis, rank, score, or positional distance has quantitative meaning.

**Figure 2:**
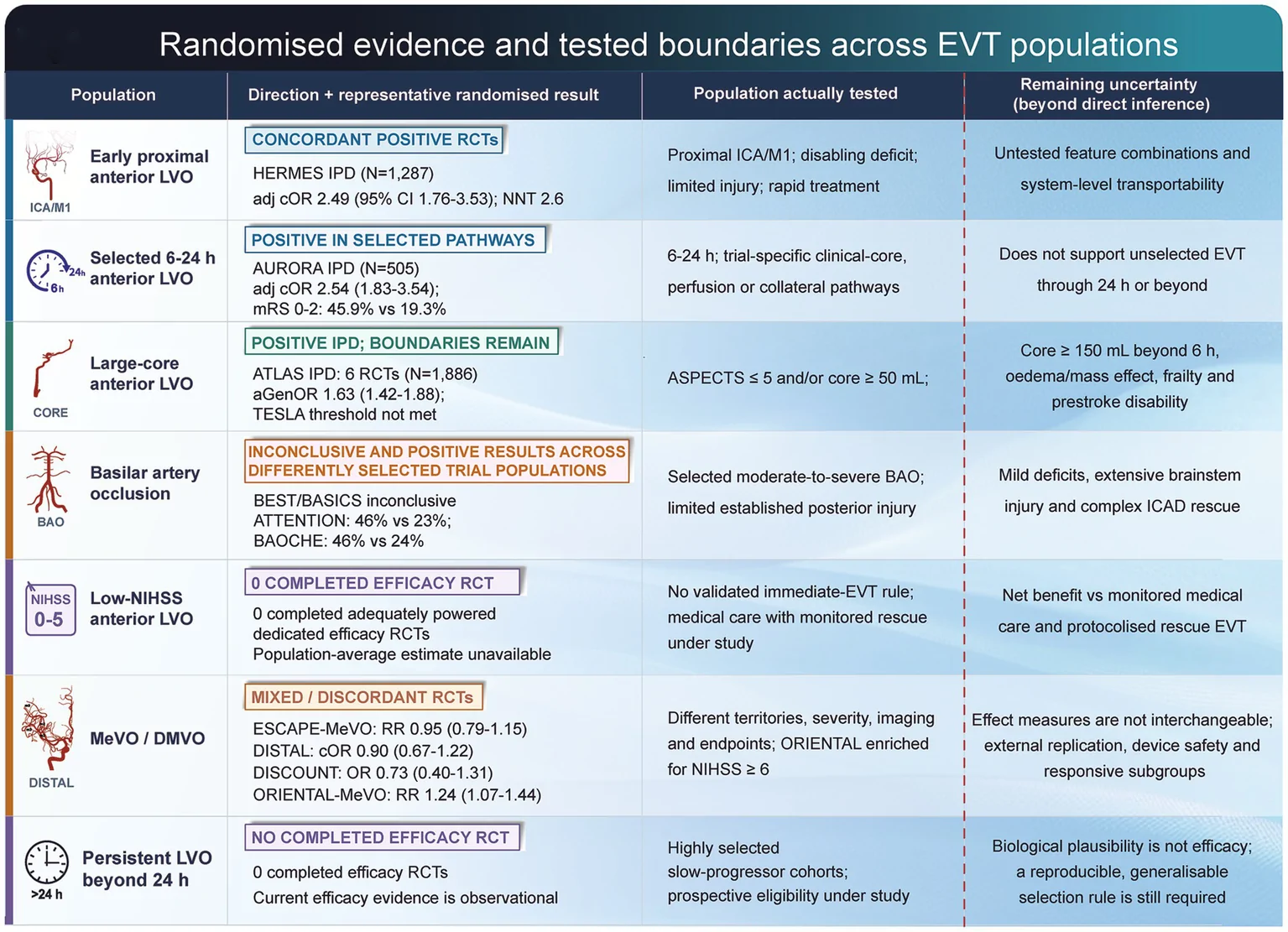
Randomised evidence and tested boundaries across EVT populations. Rows follow manuscript order and do not represent chronology, evidence rank or a maturity/heterogeneity scale. Each row reports the direction or status of completed dedicated efficacy RCT evidence, a representative effect estimate on its original outcome scale, the population boundary actually tested and the uncertainty remaining beyond direct randomised inference. Early proximal LVO is anchored by HERMES (2); selected 6–24 h anterior LVO by AURORA (14); large-core LVO by ATLAS and TESLA (21, 22); BAO by BEST, BASICS, ATTENTION, and BAOCHE (23–26); low-NIHSS LVO has no completed adequately powered dedicated efficacy RCT (41–47); MeVO/DMVO displays the non-interchangeable estimates from ESCAPE-MeVO, DISTAL, DISCOUNT, and ORIENTAL-MeVO (27–30); and treatment beyond 24 h has no completed efficacy RCT (52–60). Direction tags are author-developed, nonvalidated and nonordinal; colour is redundant with text. Absence of a completed RCT means absence of a completed efficacy estimate, not evidence of no effect. Because endpoints and effect scales differ, estimates should not be compared as a common metric. The figure is a population-level evidence-interpretation aid, not an individual prediction model or treatment rule. BAO, basilar artery occlusion; cOR, common odds ratio; EVT, endovascular thrombectomy; IPD, individual-participant data; LVO, large-vessel occlusion; MeVO/DMVO, medium/distal-vessel occlusion; NIHSS, National Institutes of Health Stroke Scale; RCT, randomised controlled trial.

## 4 Applying the framework across EVT populations

### 4.1 Early and selected 6–24 h anterior-circulation LVO

#### 4.1.1 Randomized evidence and trial eligibility

The early pivotal trials and HERMES tested rapid EVT plus standard care versus standard care alone in patients with proximal anterior-circulation LVO, meaningful neurological deficit, and limited established injury (2–7). In 1,287 participants, HERMES found a shift toward less disability with EVT (adjusted common odds ratio [cOR] 2.49, 95% CI 1.76–3.53), with a number needed to treat of 2.6 for improvement by at least one mRS category (2). DAWN enrolled 206 patients with age- and NIHSS-stratified clinical–core mismatch at 6–24 h; mean utility-weighted mRS was 5.5 versus 3.4, and mRS 0–2 occurred in 49% versus 13% (12). DEFUSE 3 enrolled 182 patients with core volume below 70 mL and perfusion mismatch at 6–16 h; the unadjusted cOR for a better 90-day mRS was 2.77, and mRS 0–2 occurred in 45% versus 17% (13). AURORA pooled 505 participants from six late-window RCTs and found an adjusted cOR of 2.54 (95% CI 1.83–3.54) (14). MR CLEAN-LATE randomized 535 participants, with 502 included in the modified intention-to-treat analysis, and reported an adjusted cOR of 1.67 (95% CI 1.20–2.32) (15).

#### 4.1.2 Baseline prognosis

Established infarct burden, age, baseline disability, NIHSS, collateral status, and treatment delay are associated with observed outcome across early- and late-window datasets (2, 12–15, 31). These features can change absolute expected gain through baseline risk even when a relative interaction is not demonstrated (8).

#### 4.1.3 Procedural feasibility and safety

Proximal anatomy and device access favor a high probability of reperfusion, but workflow delay attenuates benefit (31). Advanced imaging is useful when it reproduces a validated selection rule without materially delaying treatment (12–15). A faster computed tomography angiography (CTA)-based pathway may therefore be preferable to richer imaging when local acquisition or transfer delays would erase the value of added information (15, 31, 32).

#### 4.1.4 Treatment-effect modification and residual uncertainty

HERMES found no convincing heterogeneity across several prespecified subgroups, but this does not prove identical effects for every untested combination of features (2, 8). Time-to-treatment analyses support urgency and show attenuation with delay (31). The causal conclusion is restricted to trial-like populations: late-window benefit is evidence for specific clinical–core, perfusion, or collateral pathways, not for unselected EVT throughout 24 h or beyond (12–15).

### 4.2 Large-core anterior-circulation infarction

#### 4.2.1 Randomized evidence and trial eligibility

RESCUE-Japan LIMIT reported mRS 0–3 in 31.0% versus 12.7% [risk ratio (RR) 2.43] (16). ANGEL-ASPECT reported a generalized OR of 1.37 (17), SELECT2 a generalized OR of 1.51 (18), TENSION an adjusted cOR of 2.58 (19), and LASTE a generalized OR of 1.63 (20). TESLA used an Alberta Stroke Program Early CT Score (ASPECTS) of 2–5 based on non-contrast computed tomography (NCCT) and reported a utility-weighted mRS difference of 0.63 (95% credible interval −0.09 to 1.34), which did not meet its prespecified Bayesian superiority threshold (21). ATLAS pooled centrally adjudicated IPD from 1,886 participants in all six large-core RCTs and found a better 90-day mRS distribution with EVT (adjusted generalized OR 1.63, 95% CI 1.42–1.88), without a significant difference in symptomatic intracranial hemorrhage (1.1% versus 1.0%) (22). The source trials differed in imaging modality, core definition, and time window; none compared imaging modalities head-to-head (16–22).

#### 4.2.2 Baseline prognosis

Core volume or ASPECTS, infarct topology, edema, age, baseline function, NIHSS, and collateral status are associated with independence, survival, and extreme dependency in large-core trial populations (16–22, 33–36). In this setting, an ordinal shift away from death or severe dependency may be clinically meaningful even when mRS 0–2 remains uncommon (16–20, 22).

#### 4.2.3 Procedural feasibility and safety

Mass effect, very extensive injury, vascular access, time to reperfusion, and hemorrhagic risk affect deliverability. Any intracranial hemorrhage was more frequent with EVT in RESCUE-Japan LIMIT and ANGEL-ASPECT (16, 17), whereas ATLAS found no significant difference in symptomatic intracranial hemorrhage across six trials (22). Symptomatic-hemorrhage definitions and rates nevertheless varied; cross-trial safety comparisons must remain qualitative (20–22).

#### 4.2.4 Treatment-effect modification and residual uncertainty

The pivotal trials did not validate every imaging or clinical feature as a modifier. A meta-analysis of large-core RCT data found no significant interaction between perfusion mismatch status and EVT effect across tested outcomes (37). A secondary TENSION analysis found an interaction for quantitative net water uptake (34), whereas single-phase CTA collateral grade was prognostic without evidence of differential EVT benefit (35). LASTE and its ancillary analysis extend randomized evidence to selected patients with ASPECTS 0–2, particularly in earlier windows (20, 36); these findings should not be generalized to profound edema or mass effect, extreme core volumes, marked frailty, or late-window overlap (see Tables 1, 2).

**Table 1:** Applying the four-question treatment-response framework across major EVT populations

Population	What randomized trials tested (eligibility/evidence)	Baseline prognosis (not automatically a modifier)	Procedural feasibility and safety	Credible treatment-effect modification	Population-level clinical inference
Early proximal anterior-circulation LVO	ICA/proximal MCA occlusion; meaningful deficit; limited established injury; rapid EVT within trial window; multiple positive RCTs and HERMES IPD meta-analysis (2–7).	Age, prestroke mRS, NIHSS, ASPECTS/core burden, collaterals, and delay are prognostic (2, 31, 33).	Proximal access and high reperfusion probability favor EVT; benefit attenuates with delay; hemorrhage and vessel injury remain relevant (2–7, 31).	HERMES did not show convincing heterogeneity across prespecified subgroups, but power for many interactions was limited (2, 8).	Established average benefit in trial-like patients; treat urgently. Eligibility does not guarantee individual benefit (2–8).
Selected 6–24 h anterior-circulation LVO	DAWN: age/NIHSS–core mismatch, 6–24 h; DEFUSE 3: core <70 mL plus perfusion mismatch, 6–16 h; MR CLEAN-LATE: CTA collateral flow, 6–24 h; AURORA pooled selected late-window trials (12–15).	Core volume, mismatch, collaterals, age, baseline function, NIHSS, and delay are prognostic (12–15, 31).	Imaging should reproduce a validated pathway without material delay; CTA may be more scalable than perfusion/MRI in some systems (12–15, 31, 32).	No evidence shows that clinical–core, perfusion, and collateral rules are biologically interchangeable; absence of interaction is not proof of homogeneity (8, 12–15).	Established benefit only for prospectively selected late-window populations; not evidence for unselected EVT or treatment beyond 24 h (12–15).
Trial-defined large-core anterior-circulation stroke	RESCUE-Japan LIMIT, ANGEL-ASPECT, SELECT2, TENSION, and LASTE were positive using different ASPECTS/core and time rules; TESLA did not meet its prespecified Bayesian superiority threshold. ATLAS pooled IPD from all six RCTs and confirmed an ordinal disability benefit (16–22).	ASPECTS/core volume, topology, edema, age, prestroke mRS, NIHSS, and collaterals are prognostic (16–22, 33–36).	Mass effect, extreme injury, hemorrhagic risk, access, and delay affect deliverability; safety definitions vary (16–22).	A meta-analysis found no significant interaction between perfusion mismatch status and EVT effect; secondary TENSION data suggested an interaction for net water uptake, whereas single-phase CTA collateral grade was prognostic without evidence of differential EVT benefit. These analyses do not establish a general bedside modifier rule (34, 35, 37).	Average ordinal disability benefit is supported in trial-defined populations. LASTE extends evidence to selected ASPECTS 0–2 patients; ATLAS improves precision but does not remove uncertainty for core ≥150 mL beyond 6 h, marked edema/mass effect, frailty, or prestroke disability (16–22, 36).
Selected moderate-to-severe BAO	BEST/BASICS were neutral or inconclusive in broad populations; ATTENTION (≤12 h) and BAOCHE (6–24 h) were positive in clinically/imaging-enriched BAO (23–26).	NIHSS, PC-ASPECTS/brainstem injury, collaterals, age, and prestroke mRS are prognostic; NIHSS may underrepresent posterior symptoms (23–26, 38–40).	ICAD, perforator anatomy, and need for rescue angioplasty/stenting alter procedural and antithrombotic risk (23–26, 39, 40).	No definitive modifier rule for mild BAO, collateral grade, etiology, or rescue strategy; most subgroup analyses were underpowered (38–40).	Benefit is supported for trial-like clinically significant BAO with limited injury in experienced centers; mild BAO and extensive brainstem infarction remain uncertain (25, 26, 38–40).
Low-NIHSS anterior-circulation LVO	No completed adequately powered dedicated efficacy RCT; evidence is observational. MOSTE, STEP, and MILD-MT test immediate EVT against monitored medical/rescue pathways (41–47).	Deficit type/severity, occlusion site, eloquence, perfusion deficit, collaterals, and early-deterioration risk are prognostic/candidate enrichment features (41–47).	Because untreated prognosis may be favorable, embolization, dissection, perforation, and hemorrhage consume a larger share of possible benefit (41–47).	No validated interaction-based selection rule. Candidate features must not be presented as established treatment criteria (41–47).	Routine immediate EVT is unproven; trial enrolment or structured monitoring with protocolized rescue treatment is appropriate (41–47).
MeVO/DMVO	ESCAPE-MeVO, DISTAL, and DISCOUNT did not show average benefit in broad populations; ORIENTAL-MeVO was positive in an enriched NIHSS ≥6 population (27–30). Historical M2 evidence and contemporary synthesis/guidance require narrower interpretation (48–51).	Territory eloquence, deficit type/severity, tissue at risk, collaterals, and spontaneous/IVT recanalization are prognostic/candidate enrichment features (27–30, 49–51).	Small caliber, tortuosity, device–vessel mismatch, and perforation/SAH risk narrow the benefit–harm margin (27–30, 49–51).	No definitive interaction by territory, NIHSS, or device; cross-trial differences are hypotheses, not validated modifiers (8, 27–30, 50, 51).	Routine anatomy-based EVT is not supported. ORIENTAL-MeVO supports replication in its defined enriched population, not generalization to all distal occlusions (27–30, 50, 51).
Persistent LVO beyond 24 h	No completed efficacy RCT. Available studies are highly selected observational cohorts; LATE-MT is testing a prospective rule (52–60).	Persistent mismatch, favorable collaterals, limited injury, and clinical stability identify slow progressors and predict outcome (52–59).	Transfer and treatment delay can eliminate residual tissue; feasibility is system-dependent (52–60).	No randomized interaction evidence. Apparent benefit is vulnerable to measured and unmeasured selection (8, 54–59).	Biological plausibility justifies trials but does not establish efficacy; routine EVT beyond 24 h is unproven (52–60).

Interpretive rule: eligibility criteria identify the population studied; prognostic features predict outcome under one or both strategies; feasibility/safety features affect deliverability and harm; a treatment-effect modifier requires randomized interaction evidence on a specified outcome and effect scale. None of these categories is an individual prediction model.

ASPECTS, Alberta Stroke Program Early CT Score; BAO, basilar artery occlusion; CTA, computed tomography angiography; EVT, endovascular thrombectomy; ICA, internal carotid artery; ICAD, intracranial atherosclerotic disease; IPD, individual-participant data; IVT, intravenous thrombolysis; LVO, large-vessel occlusion; MCA, middle cerebral artery; MeVO/DMVO, medium/distal-vessel occlusion; mRS, modified Rankin Scale; NIHSS, National Institutes of Health Stroke Scale; RCT, randomized controlled trial; SAH, subarachnoid hemorrhage.

**Table 2:** Pivotal randomized trials and individual-participant-data syntheses defining major EVT populations

Population/study	Sample size (N)	Key eligibility and time window	Intervention/comparator	Primary outcome and quantitative effect	Major safety outcome(s)	Principal limitation/boundary
Early proximal anterior LVO—HERMES IPD meta-analysis (2)	1,287	Five early-window RCTs; proximal anterior-circulation occlusion; trial-specific windows, with EVT assigned within 12 h; trial-specific limited-injury criteria.	EVT + standard care vs. standard care.	Better 90-day mRS distribution: adjusted cOR 2.49 (95% CI 1.76–3.53); NNT 2.6 for ≥1-category mRS improvement.	sICH 4.4% vs. 4.3%; 90-day mortality 15.3% vs. 18.9%.	IPD meta-analysis, not one uniform trial; does not define untested large-core, mild-deficit, posterior, or late-window populations.
Early proximal anterior LVO—MR CLEAN (3)	500	Proximal anterior-circulation occlusion confirmed on vascular imaging; treatment possible within 6 h.	Intra-arterial treatment plus usual care vs. usual care alone.	Better 90-day mRS distribution: adjusted cOR 1.67 (95% CI 1.21–2.30); mRS 0–2: 32.6% vs. 19.1%.	sICH 7.7% vs. 6.4%; 90-day mortality 21.0% vs. 22.1%.	Pragmatic first positive trial; intra-arterial treatment included evolving devices, and not every assigned patient received an endovascular procedure.
Late anterior LVO—DAWN (12)	206	ICA/proximal MCA; 6–24 h; age/NIHSS-stratified clinical–core mismatch by automated CTP/DWI.	EVT + standard care vs. standard care.	Mean utility-weighted mRS 5.5 vs. 3.4; mRS 0–2: 49% vs. 13%.	sICH 6% vs. 3%; mortality 19% vs. 18%.	Highly selected clinical–core mismatch; no evidence for unselected 6–24 h treatment.
Late anterior LVO—DEFUSE 3 (13)	182	ICA/MCA; 6–16 h; core <70 mL, mismatch ratio ≥1.8, and mismatch volume ≥15 mL.	EVT + medical therapy vs. medical therapy.	Better 90-day mRS distribution: unadjusted cOR 2.77 (95% CI 1.63–4.70); mRS 0–2: 45% vs. 17%.	sICH 7% vs. 4%; mortality 14% vs. 26%.	Perfusion thresholds are a trial selection instrument, not a time-independent guarantee.
Late anterior LVO—AURORA IPD meta-analysis (14)	505	Six RCTs randomized >6 h to ≤24 h under trial-specific clinical/imaging enrichment.	EVT + medical care vs. medical care.	Better 90-day mRS distribution: adjusted cOR 2.54 (95% CI 1.83–3.54); mRS 0–2: 45.9% vs. 19.3%.	sICH 5.3% vs. 3.3%; mortality 16.5% vs. 19.3%.	Confirms benefit in pooled trial-like populations; does not make selection pathways interchangeable.
Late anterior LVO—MR CLEAN-LATE (15)	535/502 mITT	Anterior LVO, NIHSS ≥2, CTA collateral flow; 6–24 h; DAWN/DEFUSE 3-eligible patients excluded.	EVT + best medical care vs. best medical care.	Better 90-day mRS distribution: adjusted cOR 1.67 (95% CI 1.20–2.32); median mRS 3 vs. 4.	sICH 7% vs. 2%; mortality 24% vs. 30%.	Supports collateral-based selection; not absent collaterals or >24 h treatment.
Large core—RESCUE-Japan LIMIT (16)	203	ICA/M1 LVO; ASPECTS 3–5; ≤6 h, or ≤24 h with no early FLAIR change; predominantly MRI selection.	EVT + medical care vs. medical care.	mRS 0–3 at 90 days: 31.0% vs. 12.7%; RR 2.43 (95% CI 1.35–4.37).	sICH 9.0% vs. 4.9%; mortality 18.0% vs. 23.5%; any ICH 58.0% vs. 31.4%.	Japan-specific imaging/treatment context; dichotomous primary endpoint and high any-ICH rate.
Large core—ANGEL-ASPECT (17)	456	Anterior LVO; ≤24 h; ASPECTS 3–5 or core 70–100 mL under trial-specific rules.	EVT + medical management vs. medical management.	Better 90-day mRS distribution: generalized OR 1.37 (95% CI 1.11–1.69).	sICH 6.1% vs. 2.7%; mortality 21.7% vs. 20.0%; any ICH 49.1% vs. 17.3%.	Mixed imaging rules; predominantly Chinese population; increased any ICH.
Large core—SELECT2 (18)	352	ICA/M1; ≤24 h; ASPECTS 3–5 or CTP/DWI core ≥50 mL.	EVT + medical care vs. medical care.	Better 90-day mRS distribution: generalized OR 1.51 (95% CI 1.20–1.89); mRS 0–2: 20% vs. 7%.	sICH 0.6% vs. 1.1%; mortality 38.4% vs. 41.5%.	Very low ASPECTS and extreme core volumes were incompletely represented.
Large core—TENSION (19)	253	Anterior LVO; predominantly NCCT ASPECTS 3–5; ≤12 h.	EVT + medical treatment vs. medical treatment.	Better 90-day mRS distribution: adjusted cOR 2.58 (95% CI 1.60–4.15).	sICH 6% vs. 5%; mortality 40% vs. 51%.	Pragmatic ≤12 h evidence; does not establish the full 24 h or extreme-core boundary.
Large core—LASTE (20)	333/324 analyzed	Proximal anterior LVO; ASPECTS ≤5; known onset ≤6.5 h or unknown onset with DWI–FLAIR mismatch.	EVT + medical care vs. medical care.	Better 90-day mRS distribution: generalized OR 1.63 (95% CI 1.29–2.06).	sICH 9.6% vs. 5.7%; mortality 36.1% vs. 55.5%.	Predominantly early-window; stopped early after external evidence; hemorrhage signal requires context.
Large core—TESLA (21)	300	ICA/M1; NCCT ASPECTS 2–5; ≤24 h.	EVT + medical management vs. medical management.	Mean utility-weighted mRS 2.93 vs. 2.27; adjusted difference 0.63 (95% CrI − 0.09 to 1.34); prespecified Bayesian superiority threshold not met.	sICH 4.0% vs. 1.3%; mortality 35.3% vs. 33.3%.	Does not compare NCCT with advanced imaging and does not negate positive trials using different rules.
Large core—ATLAS IPD meta-analysis (22)	1,886	Six large-core RCTs; ASPECTS ≤5 and/or core volume ≥50 mL; randomization within 24 h; central imaging adjudication.	EVT + medical care vs. medical care.	Better 90-day mRS distribution: adjusted generalized OR 1.63 (95% CI 1.42–1.88); NNT 4.2 for ≥1-category mRS improvement.	sICH 1.1% vs. 1.0%; 90-day mortality 31.1% vs. 37.3%; adjusted RR for mortality 0.82 (95% CI 0.70–0.97).	IPD synthesis, not a new randomized comparison; evidence remains limited for core ≥150 mL beyond 6 h and features under-represented in source trials.
BAO—BEST (23)	131	Acute vertebrobasilar occlusion; ≤8 h; open-label; substantial crossover and poor recruitment.	EVT + standard care vs. standard care.	mRS 0–3: 42% vs. 32%; adjusted OR 1.74 (95% CI 0.81–3.74).	sICH 8% vs. 0%; mortality 33% vs. 38%.	Early termination, crossover, and under-recruitment limit causal precision.
BAO—BASICS (24)	300	BAO with broad severity spectrum; ≤6 h.	EVT + medical therapy vs. medical therapy.	mRS 0–3: 44.2% vs. 37.7%; RR 1.18 (95% CI 0.92–1.50).	sICH 4.5% vs. 0.7%; mortality 38.3% vs. 43.2%.	Broad spectrum and changing eligibility/equipoise; not directly comparable with later enriched trials.
BAO—ATTENTION (25)	340	Imaging-confirmed BAO; NIHSS ≥10; age-specific prestroke mRS and PC-ASPECTS limits; ≤12 h.	EVT + best medical care vs. best medical care.	mRS 0–3: 46% vs. 23%; adjusted RR 2.06 (95% CI 1.46–2.91).	sICH 5% vs. 0%; mortality 37% vs. 55%.	China-only; limited inference for mild BAO, extensive brainstem infarction, or major prestroke disability.
BAO—BAOCHE (26)	217	Imaging-confirmed BAO; prestroke mRS 0–1; large posterior infarction excluded; 6–24 h.	EVT + best medical care vs. best medical care.	mRS 0–3: 46% vs. 24%; adjusted RR 1.81 (95% CI 1.26–2.60).	sICH 6% vs. 1%; mortality 31% vs. 42%.	China-only; endpoint modifications and enriched population limit unrestricted extrapolation.
MeVO/DMVO—ESCAPE-MeVO (27)	530	Favourable non-invasive imaging; mostly MCA branches; ≤12 h.	EVT + usual care vs. usual care.	mRS 0–1: 41.6% vs. 43.1%; adjusted RR 0.95 (95% CI 0.79–1.15).	sICH 5.4% vs. 2.2%; mortality 13.3% vs. 8.4% (adjusted HR 1.82, 95% CI 1.06–3.12).	Neutral average effect with safety concern; does not exclude a narrower responsive population.
MeVO/DMVO—DISTAL (28)	543	Isolated non-dominant/codominant M2, M3/M4, A1–A3, or P1–P3; ≤24 h.	EVT + best medical treatment vs. best medical treatment.	90-day mRS shift: cOR 0.90 (95% CI 0.67–1.22; *p* = 0.50).	sICH 5.9% vs. 2.6%; mortality 15.5% vs. 14.0%.	Heterogeneous territories and mild median severity; subgroup benefit unproven.
MeVO/DMVO—DISCOUNT (29)	244	Primary isolated distal M2/M3, ACA/PCA occlusion; ≤8 h, or ≤24 h without FLAIR hyperintensity.	EVT + medical treatment vs. medical treatment alone.	mRS 0–2: 62% vs. 68%; OR 0.73 (95% CI 0.40–1.31; *p* = 0.29); adjusted absolute difference −6.8% (95% CI − 19.4 to 5.7).	sICH 11% vs. 3% among patients who did versus did not undergo thrombectomy (as-treated safety comparison; *p* = 0.008); mortality 6% vs. 8%.	Stopped early for futility and increased sICH; 19% assigned to EVT did not undergo thrombectomy; subgroup inference limited.
MeVO—ORIENTAL-MeVO (30)	563	Primary MeVO; prestroke mRS 0–2; NIHSS ≥6; favorable imaging; ≤24 h.	EVT + medical management vs. medical management.	Proportional-odds assumption violated; prespecified fallback mRS 0–2: 58.6% vs. 46.6%; adjusted RR 1.24 (95% CI 1.07–1.44).	sICH 4.7% vs. 2.2% (adjusted RR 2.21, 95% CI 0.87–5.63); mortality 11.1% vs. 10.2%.	Enriched Chinese population; requires external replication and should not be generalized to all distal occlusions.

Percentages are EVT plus best medical care versus best medical care alone unless stated otherwise. Safety definitions differed across trials; comparisons of sICH or any ICH should therefore remain qualitative. Sample-size pairs denote randomized/primary-analysis populations. ATLAS is an IPD synthesis of six source RCTs rather than a separate randomized comparison.

ACA, anterior cerebral artery; ASPECTS, Alberta Stroke Program Early CT Score; BAO, basilar artery occlusion; cOR, common odds ratio; CrI, credible interval; CTA, computed tomography angiography; CTP, CT perfusion; DWI, diffusion-weighted imaging; EVT, endovascular thrombectomy; FLAIR, fluid-attenuated inversion recovery; HR, hazard ratio; ICA, internal carotid artery; ICH, intracranial hemorrhage; IPD, individual-participant data; ITT, intention to treat; LVO, large-vessel occlusion; MCA, middle cerebral artery; MeVO/DMVO, medium/distal-vessel occlusion; mITT, modified intention-to-treat; mRS, modified Rankin Scale; NCCT, non-contrast CT; NIHSS, National Institutes of Health Stroke Scale; NNT, number needed to treat; OR, odds ratio; PC-ASPECTS, posterior-circulation ASPECTS; RCT, randomized controlled trial; RR, risk ratio; sICH, symptomatic intracranial hemorrhage.

### 4.3 Basilar artery occlusion

#### 4.3.1 Randomized evidence and trial eligibility

BAO has a distinct natural history and should not be pooled conceptually with anterior-circulation LVO. BEST reported mRS 0–3 in 42% versus 32% (adjusted OR 1.74, 95% CI 0.81–3.74) (23), and BASICS reported 44.2% versus 37.7% (RR 1.18, 95% CI 0.92–1.50) (24). ATTENTION then reported mRS 0–3 in 46% versus 23% (adjusted RR 2.06) (25), while BAOCHE reported 46% versus 24% (adjusted RR 1.81) in the 6–24 h window (26). The later positive trials used more clinically and radiologically enriched populations than the earlier neutral or inconclusive trials (23–26).

#### 4.3.2 Baseline prognosis

Baseline NIHSS, posterior-circulation ASPECTS (PC-ASPECTS), brainstem infarct burden, collateral status, age, and prestroke function are associated with outcome in BAO trial populations (38–40). NIHSS-based thresholds may incompletely represent disabling posterior-circulation syndromes, so a low total score should not be equated automatically with a non-disabling presentation (39, 40).

#### 4.3.3 Procedural feasibility and safety

Occlusion mechanism, intracranial atherosclerotic disease, perforator anatomy, and the possible need for angioplasty or rescue stenting can alter procedural risk and antithrombotic exposure (24–26, 39). These factors affect deliverability but are not automatically treatment-effect modifiers.

#### 4.3.4 Treatment-effect modification and residual uncertainty

Current evidence supports EVT for trial-like, clinically significant BAO with limited established posterior-circulation injury in experienced systems (25, 26, 38–40). Evidence remains weaker for NIHSS 6–9, extensive brainstem infarction, major prestroke disability, and cases requiring complex rescue strategies (38–40). The later positive trials refine the population; they do not make BEST or BASICS irrelevant (26).

### 4.4 Low-NIHSS LVO

#### 4.4.1 Randomized evidence and trial eligibility

No completed, adequately powered dedicated efficacy RCT has established routine immediate EVT for anterior-circulation LVO with NIHSS 0–5. Available cohorts and meta-analyses are confounded by physician selection, rescue thrombectomy, and heterogeneous definitions of a disabling deficit (41–44). MOSTE, STEP, and MILD-MT are designed to compare immediate EVT with monitored medical management and protocolized rescue pathways (45–47).

#### 4.4.2 Baseline prognosis

Occlusion site, threatened eloquent territory, collateral status, perfusion deficit, fluctuating symptoms, and early neurological deterioration risk are prognostic or candidate enrichment variables in low-NIHSS LVO (41–43). A low NIHSS is not equivalent to low disability, particularly for aphasia, neglect, or dominant-hand weakness (43, 44).

#### 4.4.3 Procedural feasibility and safety

When untreated prognosis is relatively favorable, procedural embolization, dissection, perforation, and hemorrhage consume a larger proportion of the possible benefit. The relevant comparator in ongoing trials is high-quality monitoring with protocolized rescue EVT rather than passive non-treatment (44–46).

#### 4.4.4 Treatment-effect modification and residual uncertainty

No proposed variable combination has been validated as a bedside treatment-effect rule (45, 46). Immediate EVT should therefore be tested within prespecified trial pathways, with monitored medical management and protocolized rescue treatment retained as the comparator (45–47).

### 4.5 MeVO/DMVO

#### 4.5.1 Randomized evidence and trial eligibility

The major MeVO/DMVO trials enrolled different anatomical territories, deficit severities, imaging profiles, time windows, and device strategies; they should not be generalized as one homogeneous distal-occlusion population. ESCAPE-MeVO enrolled 530 patients within 12 h with favorable imaging and found mRS 0–1 in 41.6% with EVT versus 43.1% with usual care (adjusted RR 0.95, 95% CI 0.79–1.15), with mortality of 13.3% versus 8.4% (adjusted hazard ratio 1.82, 95% CI 1.06–3.12) and symptomatic intracranial hemorrhage (sICH) of 5.4% versus 2.2% (27). DISTAL enrolled 543 participants with isolated non-dominant or codominant M2, M3/M4, A1–A3, or P1–P3 occlusion within 24 h; the cOR for a better mRS was 0.90 (95% CI 0.67–1.22), with sICH of 5.9% versus 2.6% and mortality of 15.5% versus 14.0% (28). DISCOUNT randomized 244 patients and stopped early after a recommendation based on lack of efficacy and increased sICH; mRS 0–2 occurred in 62% versus 68% (OR 0.73, 95% CI 0.40–1.31), and sICH among patients who did versus did not undergo thrombectomy was 11% versus 3% in an as-treated safety comparison (29). These three trials do not support routine anatomy-based EVT across broad MeVO/DMVO populations.

ORIENTAL-MeVO enrolled 563 patients in China with NIHSS at least 6, favorable imaging, and treatment within 24 h. The proportional-odds assumption for the planned ordinal model was violated; the prespecified fallback endpoint of mRS 0–2 favored EVT (58.6% versus 46.6%; adjusted RR 1.24, 95% CI 1.07–1.44), while sICH was 4.7% versus 2.2% (adjusted RR 2.21, 95% CI 0.87–5.63) (30). Before these dedicated trials, a HERMES analysis suggested benefit for a selected M2 subgroup (48); however, contemporary imaging guidance emphasizes variability in distal-occlusion definitions and ascertainment (49), and pooled RCT evidence is neutral overall with a safety signal (50). The 2026 Society of Vascular and Interventional Neurology (SVIN) focused guideline does not support routine EVT for broadly defined DMVO, while allowing careful case-based consideration for selected disabling dominant-M2 occlusion (51).

#### 4.5.2 Baseline prognosis

Territory eloquence, deficit type and severity, tissue at risk, collateral status, and spontaneous or thrombolysis-mediated recanalization are candidate prognostic or enrichment variables rather than validated modifiers (27–30, 49–51). Broad mRS thresholds may underrepresent domain-specific disability, so future distal-occlusion trials should include outcomes relevant to language, dexterity, vision, cognition, and participation.

#### 4.5.3 Procedural feasibility and safety

Small caliber, tortuosity, distal access, device–vessel mismatch, and perforator or subarachnoid-hemorrhage risk narrow the benefit–harm margin (27–30, 49–51). Higher angiographic reperfusion rates cannot substitute for a demonstrated patient-centered benefit, which was absent in the broad ESCAPE-MeVO, DISTAL, and DISCOUNT comparisons (27–29).

#### 4.5.4 Treatment-effect modification and residual uncertainty

None of the four dedicated trials was powered to establish definitive interactions by territory, NIHSS, or device (8, 27–30). Discordant overall results may reflect population enrichment, outcome definition, reperfusion success, intravenous thrombolysis use, device strategy, and chance; pooled and guideline interpretations do not validate any of these as a bedside modifier (50, 51). These hypotheses require prospective replication rather than cross-trial subgroup comparison.

### 4.6 Treatment beyond 24 h

#### 4.6.1 Randomized evidence and trial eligibility

Biological plausibility exists because a minority of patients retain collateral-supported tissue and a target-mismatch profile beyond 24 h (52, 53). Comparative observational cohorts and meta-analysis report associations between EVT and better outcomes in highly selected slow progressors (54–56). Other registries describe selected EVT-treated cohorts without a randomized non-EVT contrast (57, 58), while TRACK-LVO Late provides an additional multicenter comparative cohort (59). No completed efficacy RCT has established routine EVT beyond 24 h. LATE-MT is testing a prospective rule against a concurrent comparator (60).

#### 4.6.2 Baseline prognosis

Persistent mismatch, favorable collaterals, limited established injury, and clinical stability identify an unusually selected group and are associated with outcome regardless of treatment assignment (52–59).

#### 4.6.3 Procedural feasibility and safety

These same variables influence whether transfer and EVT can occur before residual tissue is lost; access, transfer delay, and late deterioration make deliverability highly system-dependent (55–60).

#### 4.6.4 Treatment-effect modification and residual uncertainty

These observations do not establish efficacy. A patient who remains stable, reaches an EVT center, undergoes advanced imaging, and is offered treatment after 24 h has passed multiple measured and unmeasured selection filters (55–59). Statistical adjustment cannot reproduce randomization (8). Biological plausibility is therefore a rationale for prospective testing, not evidence that EVT improves outcome (52, 53). Treatment beyond 24 h should be described as evidence-uncertain and, where feasible, evaluated within randomized trials rather than presented as an established extension of the EVT time window.

## 5 Interpreting post-treatment reperfusion and outcome

The term “futile reperfusion” is widely used for poor functional outcome despite angiographic success (9, 61, 62), but it can imply absence of benefit that treated-patient data cannot establish. We therefore use the neutral description of poor outcome despite successful reperfusion. A treated patient with mRS 4 may still have avoided mRS 6 because the untreated counterfactual is unknown. Conversely, angiographic success can coexist with vessel injury, hemorrhage, or absent tissue-level recovery (63–66).

Reported frequencies depend on definitions. In the ANGEL-ACT registry, 51.8% of 1,158 anterior-circulation LVO patients met the study definition: successful reperfusion was mTICI 2b–3 and poor outcome was mRS 3–6 at 90 days (61). In a 99-patient late-window cohort, 51.5% met the same mTICI 2b–3 and 90-day mRS 3–6 thresholds (62). These similar percentages do not make the cohorts interchangeable and do not estimate an individual causal effect.

Angiographic reperfusion is an important procedural endpoint but an incomplete surrogate for tissue reperfusion and patient-centered recovery. Different mTICI thresholds, adjudication methods, and clinical endpoint definitions can materially alter apparent success and cross-study comparability (63, 64). No-reflow, distal embolization, re-occlusion, edema, hemorrhagic transformation, and systemic complications may help explain the dissociation, but much mechanistic evidence remains observational or preclinical (65, 66). First-pass effect and device-pass number are post-randomization variables associated with outcome; they do not identify pretreatment modifiers (67, 68).

A recent viewpoint has argued that futility and harm terminology can describe population-level benefit–harm thresholds (9). We agree that endpoint choice matters, but classifying an individual treated patient as having received no benefit still requires an unobserved untreated counterfactual (8, 9). We therefore use poor outcome despite successful reperfusion for treated-patient outcomes and reserve causal claims for comparative evidence. The clinically relevant trial question remains whether assignment to EVT changes a patient-centered outcome compared with the appropriate non-EVT strategy.

## 6 Discussion

The framework reorganizes EVT evidence around evidentiary roles rather than around a catalog of technologies or trial names. Its central claim is deliberately limited: it is a population-level interpretation tool, not a validated algorithm for predicting an individual response. For every population, the same sequence applies—identify the randomized population, describe prognosis under either strategy, assess whether the procedure can be delivered safely and promptly, and then ask whether randomized evidence supports effect modification.

This organization clarifies the factual evidence matrix in Figure 2. Early proximal anterior-circulation LVO and selected 6–24 h LVO have concordant causal evidence in trial-defined populations (2–7, 12–15). The ATLAS IPD analysis confirms an overall large-core benefit across six RCTs while preserving source-trial selection boundaries, and TESLA remains important because it did not meet its prespecified superiority threshold (16–22). BAO evidence progressed from neutral or inconclusive trials to positive enriched-population trials and guideline-supported selection boundaries (23–26, 38–40). Low-NIHSS LVO lacks a completed dedicated efficacy RCT (41–47), and treatment beyond 24 h remains supported only by selected observational evidence and an ongoing RCT protocol (52–60). MeVO/DMVO evidence is population-dependent: three broad-population trials did not show average benefit, whereas ORIENTAL-MeVO was positive in an enriched population (27–30, 48–51).

The framework also limits overinterpretation of imaging. ASPECTS and estimated core volume describe established injury; perfusion mismatch and collaterals estimate residual tissue at risk; CTA defines the occlusion and vascular route (12–21, 33, 35). These measures can determine trial eligibility, carry prognostic information, or affect feasibility. They become treatment-effect modifiers only when the randomized comparison supports an interaction on a stated outcome scale (8, 34, 35, 37). The same evidentiary discipline applies to age, NIHSS, etiology, and transfer pathway.

Future trials should prespecify the causal contrast, primary outcome, and effect scale; distinguish enrichment variables from hypothesized modifiers; and report relative and absolute effects. Interaction analyses require adequate power, multiplicity control, and replication (8). Low-NIHSS studies should compare immediate EVT with monitored medical management and protocolized rescue treatment (45–47). MeVO/DMVO studies should harmonize anatomical definitions, disabling-deficit criteria, endpoints, and device-safety reporting (49–51). Beyond-24 h trials require concurrent randomized controls and prospective eligibility rules to overcome slow-progressor selection (52–60). BAO studies should address mild but disabling syndromes and extensive brainstem injury (38–40), while large-core studies should clarify core ≥150 mL beyond 6 h, profound edema or mass effect, frailty, prestroke disability, and late-window overlap despite the more precise overall ATLAS estimate (16–22, 36).

Transportability is separate from efficacy. A trial selection rule can reproduce biological enrichment yet perform differently when imaging speed, transfer time, operator experience, or thrombectomy capacity changes; delays from hospital arrival to EVT are associated with worse patient-reported outcomes (31, 69). Implementation studies should therefore report treatment delay, access, reperfusion, complications, and patient-centered outcomes. Artificial intelligence tools may standardize image interpretation or alerts, but they support workflow rather than estimate treatment effect, and their effect depends on team engagement (70, 71).

A 2026 focused guideline from the Society of Vascular and Interventional Neurology reaches the same population-specific conclusion: routine EVT for broadly defined DMVO is not supported by current evidence, while treatment may remain reasonable in carefully selected disabling dominant-M2 occlusion (51). This distinction reinforces the need to avoid extrapolation across distal territories.

This review has limitations. Study selection was targeted and purposive, not exhaustive, and no formal risk-of-bias instrument or quantitative synthesis was applied. Cross-trial comparisons are indirect because eligibility criteria, outcome scales, and safety definitions differed. Some modifier analyses were exploratory and underpowered. The framework itself has face validity and causal coherence but has not been prospectively tested as a clinical decision aid; it should not be used as one.

## 7 Conclusion

EVT evidence is most interpretable when trial eligibility, prognosis, procedural feasibility and safety, post-treatment technical measures, observed outcome, and causal treatment effect remain distinct (8, 9). RCTs establish average benefit in defined populations; they do not reveal an individual counterfactual. Early and selected late-window anterior-circulation LVO have supportive randomized evidence (2–7, 12–15), as do trial-defined large-core infarction and selected moderate-to-severe BAO populations within their tested boundaries (16–26, 38–40). Low-NIHSS LVO and treatment beyond 24 h remain unproven (41–47, 52–60), while MeVO/DMVO evidence is population-dependent and does not support routine EVT across all distal occlusions (27–30, 48–51).

The treatment-response framework adds value by applying one explicit evidentiary sequence to each population. Its purpose is not to label a treated outcome as beneficial or futile, but to clarify what has been tested, which features are merely prognostic or procedural, whether credible treatment-effect modification exists, and where new randomized comparisons are required (8, 9).
