# Supplementary material for: A treatment-response framework for interpreting endovascular thrombectomy evidence: established practice, boundary expansion and unresolved frontiers
Source: Front Neurol. 2026 Sep 22;17:1951552. doi: 10.3389/fneur.2026.1951552 (PMC13639571; doi:10.3389/fneur.2026.1951552)
Supplement: Supplementary file 1 [file Supplementary_file_1.docx]

Supplementary Material

## Supplementary Methods

This structured narrative review used targeted and purposive study selection rather than exhaustive systematic screening. The revision update was completed on 15 August 2026. PubMed/MEDLINE was the primary bibliographic database. Backward and forward citation tracking began with pivotal randomised controlled trials (RCTs), individual-participant-data (IPD) meta-analyses, population-specific guidelines and recent high-quality reviews. Targeted publisher and trial-registry searches were used to identify online-first articles and ongoing studies that were not yet fully indexed.

The review did not use PRISMA screening, calculate database yield or deduplication counts, prepare a flow diagram, apply a formal risk-of-bias instrument or conduct new quantitative pooling. The exact PubMed strings below are the date-stamped searches used for the revision update; they replace the previous description of only the “core logic” of historical searches.

## Supplementary Table S1. Exact PubMed/MEDLINE search strategies used for the revision update

**Common block used in every module**

("Thrombectomy"[Mesh] OR thrombectom*[tiab] OR "endovascular treatment"[tiab] OR "endovascular therapy"[tiab]) AND ("Stroke"[Mesh] OR "ischemic stroke"[tiab] OR "ischaemic stroke"[tiab])

| **Module** | **Exact PubMed/MEDLINE query appended to the common block** | **Last run** | **Purpose** |
| --- | --- | --- | --- |
| 1. Late-window and imaging selection | AND ("late window"[tiab] OR "extended window"[tiab] OR "6-24 hour"[tiab] OR "6 to 24 hour"[tiab] OR perfusion[tiab] OR mismatch[tiab] OR collateral*[tiab] OR ASPECTS[tiab] OR DAWN[tiab] OR "DEFUSE 3"[tiab] OR AURORA[tiab] OR "MR CLEAN-LATE"[tiab]) | 15 Aug 2026 | Pivotal late-window RCTs, IPD meta-analysis, imaging-selection studies and late-window guidelines. |
| 2. Large-core infarction | AND ("large core"[tiab] OR "large ischemic core"[tiab] OR "large ischaemic core"[tiab] OR "large infarct"[tiab] OR "RESCUE-Japan LIMIT"[tiab] OR "ANGEL-ASPECT"[tiab] OR SELECT2[tiab] OR TENSION[tiab] OR LASTE[tiab] OR TESLA[tiab]) | 15 Aug 2026 | Large-core RCTs, trial-level meta-analyses and modifier analyses. |
| 3. Basilar artery occlusion | AND ("basilar artery occlusion"[tiab] OR BAO[tiab] OR vertebrobasilar[tiab] OR BEST[tiab] OR BASICS[tiab] OR ATTENTION[tiab] OR BAOCHE[tiab]) | 15 Aug 2026 | BAO RCTs, long-term follow-up, posterior-circulation imaging and guidelines. |
| 4. Low-NIHSS LVO | AND (("low NIHSS"[tiab] OR "minor stroke"[tiab] OR "mild stroke"[tiab] OR "nondisabling stroke"[tiab] OR "non-disabling stroke"[tiab]) AND ("large vessel occlusion"[tiab] OR LVO[tiab] OR "middle cerebral artery"[tiab])) | 15 Aug 2026 | Comparative cohorts, syntheses and dedicated trial protocols/registrations. |
| 5. MeVO/DMVO | AND ("medium vessel occlusion"[tiab] OR "medium-vessel occlusion"[tiab] OR "distal vessel occlusion"[tiab] OR "distal medium vessel occlusion"[tiab] OR MeVO[tiab] OR DMVO[tiab] OR "ESCAPE-MeVO"[tiab] OR DISTAL[tiab] OR DISCOUNT[tiab] OR "ORIENTAL-MeVO"[tiab]) | 15 Aug 2026 | Final reports of ESCAPE-MeVO, DISTAL, DISCOUNT and ORIENTAL-MeVO plus directly relevant syntheses. |
| 6. Reperfusion endpoints and poor outcome | AND (reperfusion[tiab] OR recanalization[tiab] OR recanalisation[tiab] OR "no-reflow"[tiab] OR "futile reperfusion"[tiab] OR "futile recanalization"[tiab] OR "futile recanalisation"[tiab] OR mTICI[tiab] OR eTICI[tiab]) | 15 Aug 2026 | Definitions, incidence studies, no-reflow mechanisms and endpoint-methodology papers. |
| 7. Workflow and evidence translation | AND (workflow[tiab] OR transfer[tiab] OR "door to puncture"[tiab] OR "artificial intelligence"[tiab] OR automated[tiab] OR guideline[tiab] OR "treatment effect"[tiab] OR interaction[tiab] OR prognostic[tiab]) | 15 Aug 2026 | Treatment delay, imaging pathway, implementation, guidelines and treatment-effect interpretation. |

### Targeted online-first update

Recent and online-first reports individually verified during revision included: ATLAS (10.1016/S0140-6736(26)00876-7), DISCOUNT (10.1001/jama.2026.8977), ORIENTAL-MeVO (10.1056/NEJMoa2514120), the 2026 AHA/ASA acute ischaemic stroke guideline (10.1161/STR.0000000000000513) and its published correction, the 2026 SVIN focused MeVO/DMVO guideline (10.1161/SVIN.125.002314), and the three endpoint-methodology articles discussed in response to Reviewer 2 (10.1161/STROKEAHA.125.053226; 10.1007/s00234-026-04101-w; Journal of Vascular Diseases 2026;5:18).

## Supplementary Table S2. Selection, extraction and verification rules

| **Review step** | **Operational rule** |
| --- | --- |
| Title/abstract assessment | Retain records that directly informed trial eligibility, baseline prognosis, procedural feasibility/safety, average treatment effect, treatment-effect modification, endpoint interpretation or external-validity boundaries. |
| Full-text prioritisation | Prefer primary RCT reports; use IPD meta-analyses and current guidelines to integrate trial families; use secondary analyses only for a specific modifier, endpoint or implementation question. |
| Exclusions | Case reports; purely technical records without clinical-selection relevance; duplicate reports without new information; non-English reports without unique landmark evidence. |
| Observational evidence | Include when randomised evidence was absent or when describing prognosis/mechanism; label as observational and avoid causal efficacy claims. |
| Quantitative extraction | Extract study N, population, time window, selection rule, intervention/comparator, primary endpoint, principal effect estimate, sICH and mortality when reported. |
| First reviewer | Performed extraction into the trial matrix and mapped each citation to the adjacent claim. |
| Second reviewer | Independently checked the final selected evidence set, numerical fields and claim–citation mapping against the primary article or indexed abstract. |
| Disagreement resolution | Resolve by discussion and consensus; narrow, move or remove a citation if it did not support the exact population, timing, comparator, outcome or effect claimed. |
| Treatment-effect modifier rule | Require randomised treatment-by-variable interaction evidence on a stated outcome and effect scale; do not infer modification from prognosis within one arm or from significance in one subgroup but not another. |
| Review limitations | No exhaustive duplicate screening, PRISMA flow diagram, formal risk-of-bias scoring or new meta-analysis. |

## Supplementary Table S3. Operational basis for the population-specific evidence descriptors in Figure 2

The evidence-direction descriptions are author-developed, nonvalidated and nonordinal. They do not represent chronology, a maturity or heterogeneity score, an individual prediction model or a treatment rule. Representative estimates remain on their original trial endpoints and effect scales and should not be compared as one common metric. ‘No completed efficacy RCT’ denotes absence of a completed randomised estimate, not evidence of no effect.

| **Population** | **Evidence-direction description shown in Figure 2** | **Operational basis** |
| --- | --- | --- |
| Early proximal anterior LVO | Concordant positive RCTs | Multiple pivotal RCTs and HERMES IPD show benefit in rapidly treated, trial-defined proximal anterior-circulation LVO. |
| Selected 6–24 h anterior LVO | Positive in selected pathways | Completed RCTs/IPD show benefit within clinical–core, perfusion or collateral selection pathways; these pathways are not interchangeable and do not extend evidence beyond 24 h. |
| Large-core anterior LVO | Positive IPD; boundaries remain | ATLAS integrates six RCTs and confirms an overall ordinal benefit; source-trial ASPECTS/core/window rules differed and TESLA did not meet its prespecified superiority threshold. |
| Basilar artery occlusion | Inconclusive and positive results across differently selected trial populations | BEST and BASICS were inconclusive, whereas ATTENTION and BAOCHE were positive in differently selected trial populations. Differences in eligibility, severity, imaging criteria, time window and trial conduct preclude attributing the cross-trial results to any single explanatory modifier. |
| Low-NIHSS anterior LVO | No completed efficacy RCT | No completed adequately powered dedicated RCT provides a population-average efficacy estimate; ongoing trials compare immediate EVT with monitored medical care and rescue pathways. |
| MeVO/DMVO | Mixed or discordant RCTs | ESCAPE-MeVO, DISTAL and DISCOUNT did not show average functional benefit, whereas ORIENTAL-MeVO was positive in an enriched population; endpoints/effect scales are not interchangeable. |
| Persistent LVO beyond 24 h | No completed efficacy RCT | Current efficacy evidence is observational; biological plausibility motivates randomisation but does not establish treatment benefit. |

**Abbreviations:** ASPECTS, Alberta Stroke Program Early CT Score; BAO, basilar artery occlusion; DMVO, distal medium-vessel occlusion; EVT, endovascular thrombectomy; IPD, individual-participant data; LVO, large-vessel occlusion; MeVO, medium-vessel occlusion; NIHSS, National Institutes of Health Stroke Scale; PRISMA, Preferred Reporting Items for Systematic Reviews and Meta-Analyses; RCT, randomised controlled trial; sICH, symptomatic intracranial haemorrhage.
